# Testing Usability and Acceptability of a Web Application to Promote Physical Activity (iCanFit) Among Older Adults

**DOI:** 10.2196/humanfactors.3787

**Published:** 2014-10-13

**Authors:** Yan Hong, Daniel Goldberg, Deborah Vollmer Dahlke, Marcia G Ory, Jessica S Cargill, Rachel Coughlin, Edgar Hernandez, Debra K Kellstedt, S Camille Peres

**Affiliations:** ^1^ School of Public Health Texas A&M Health Science Center College Station, TX United States; ^2^ Department of Geography Texas A&M University College Station, TX United States; ^3^ Department of Computer Science and Engineering Texas A&M University College Station, TX United States; ^4^ DVD Associates Austin, TX United States; ^5^ St Joseph Regional Health Center Bryan, TX United States; ^6^ Environmental Geosciences Program Texas A&M University College Station, TX United States

**Keywords:** physical activity, mobile health, older adults, usability testing, user experience

## Abstract

**Background:**

Most older Americans do not exercise regularly and many have chronic conditions. Among an increasing number of fitness mobile and Web apps, few are designed for older adults with chronic conditions despite high ownership rates of mobile tools and Internet access in this population. We designed a mobile-enabled Web app, iCanFit, to promote physical activity in this population.

**Objective:**

This study aimed to test the usability and acceptability of iCanFit among older adults in a community setting.

**Methods:**

A total of 33 older adults (aged 60 to 82 years) were recruited from communities to test iCanFit. Of these 33, 10 participants completed the usability testing in a computer room of a senior community center. A research assistant timed each Web application task and observed user navigation behavior using usability metrics. The other 23 participants used the website on their own devices at home and provided feedback after 2-3 weeks by completing a user-experience survey assessing ease of use, helpfulness, and satisfaction with iCanFit.

**Results:**

Participants completed all 15 tasks on the iCanFit site in an average of 31 (SD 6.9) minutes; some tasks required more time or needed assistance. Participants’ comments were addressed to improve the site’s senior friendliness and ease of use. In the user-experience survey, participants reported high levels of usefulness and satisfaction. More than 56% (13/23) of participants indicated they would continue using the program and recommend it to their families or friends.

**Conclusions:**

Testing usability and acceptability is a very important step in developing age-appropriate and user-friendly Web apps, especially for older adults. Testing usability and acceptability in a community setting can help reveal users’ experiences and feedback in a real-life setting. Our study suggested that older adults had a high degree of acceptance of iCanFit and could use it easily. The efficacy trial of iCanFit is currently underway.

## Introduction

As of January 2014, 87% of American adults have used the Internet. Even among older adults, 88% of those aged 50 to 64 years are online and more than 57% of those older than 64 years are online [1]. More than 58% of American adults own a smartphone and 42% own a tablet computer [1]. With such ubiquity of Internet access and high ownership rates of mobile tools, mobile health programs have gained increasing popularity with thousands of Web and mobile apps available for fitness and healthy living.

Approximately 13% of the American population is older than 65 years, and by 2030 older adults will account for 20% of the US population [2]. In addition, more than two-thirds of older Americans have multiple chronic conditions and medical treatment for this population accounts for 66% of the country’s health care budget [3]. Regular physical activity can prevent many chronic diseases and significantly improve quality of life for those with chronic conditions. Yet, more than 80% of older adults do not meet the guidelines of regular physical activity [4]; a recent nationwide study showed that less than 10% of adults report being active as assessed by accelerometers [5]. An urgent need exists to implement innovative and cost-effective interventions to promote healthy lifestyles among older adults.

National surveys suggest that older adults who use the Internet are more likely to seek health information online [6,7]. However, among the thousands of online apps and mobile tools available to promote physical activity, few are designed or marketed for older adults [8]. Goldberg and colleagues commented on the gap between needs and availability, stating: “The question now is not whether the public is ready for eHealth information, but whether eHealth information is ready to meet the public’s expectation” [9].

**Figure 1:**
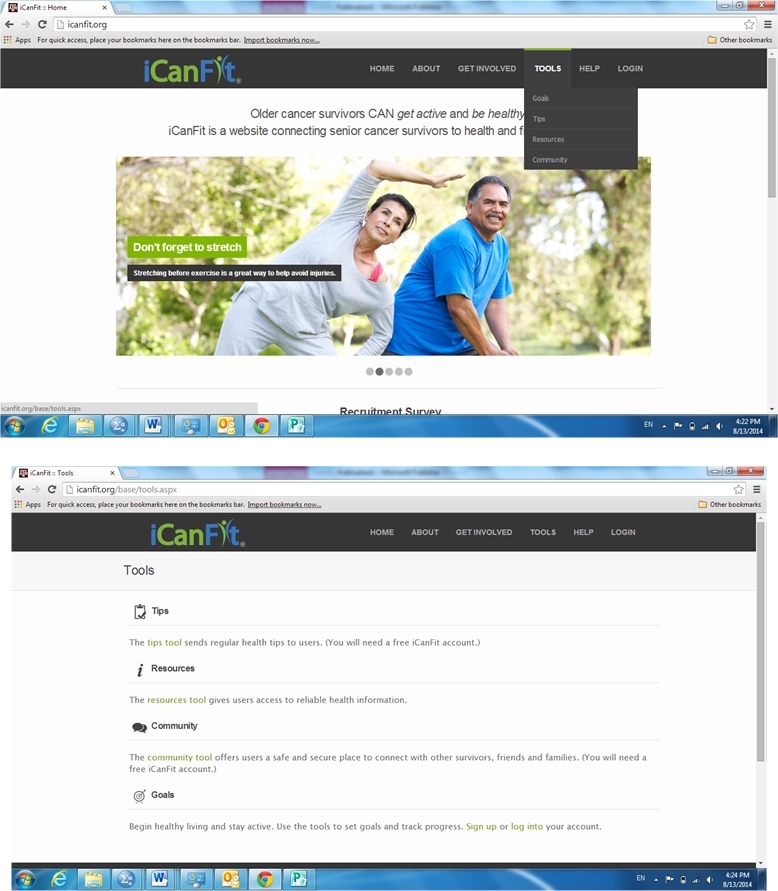
Screenshots of iCanFit.

**Figure 2:**
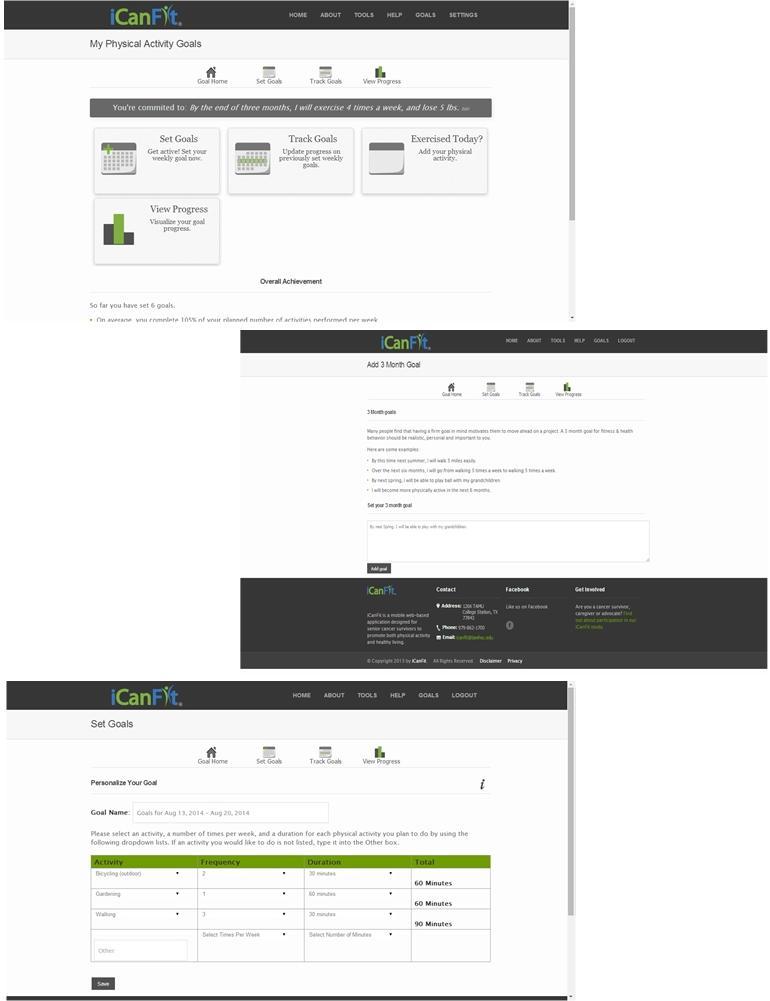
Goals function in iCanFit.

**Figure 3:**
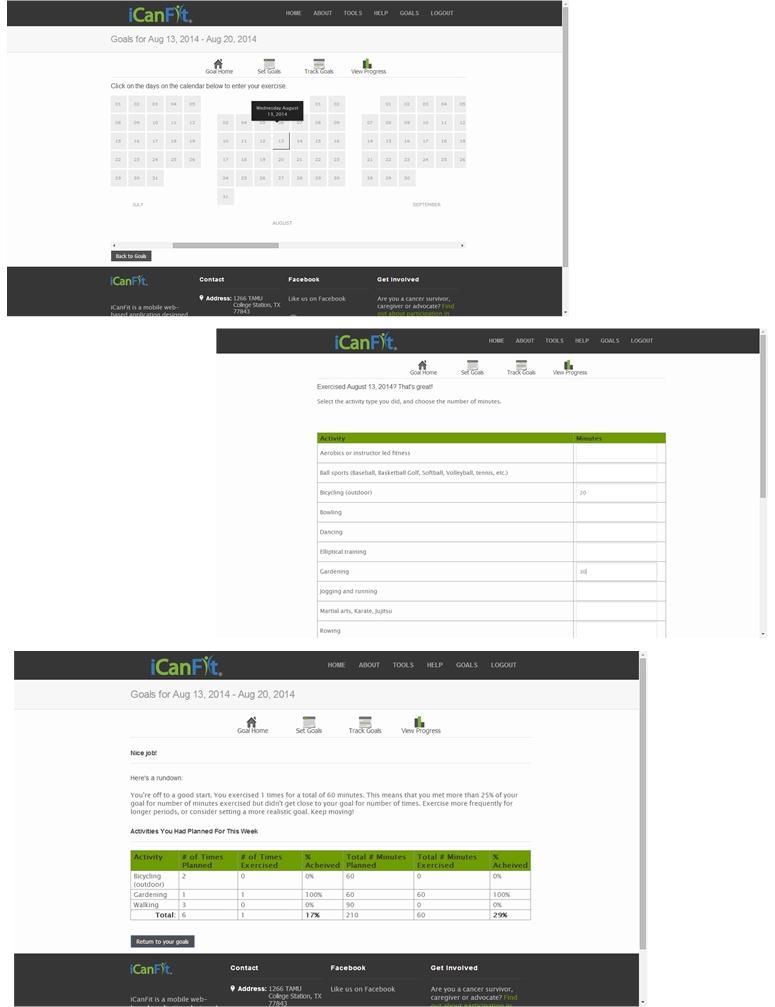
Entering, tracking, and receiving feedback of physical activity on iCanFit.

**Figure 4:**
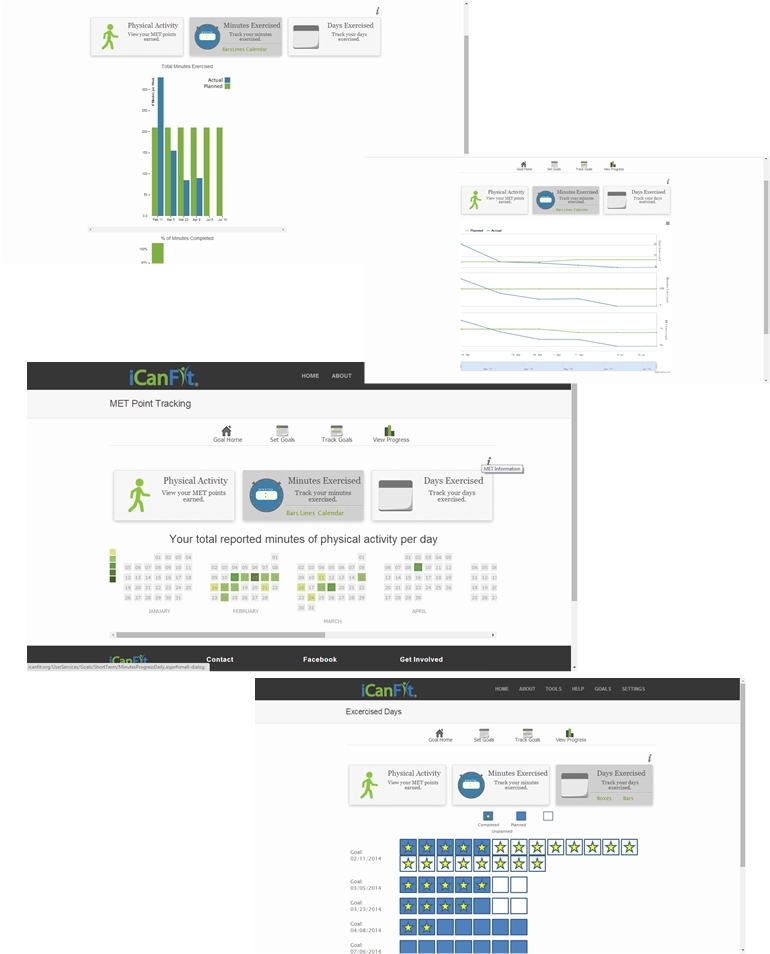
Different modes of View Progress on iCanFit.

In-line with efforts to promote physical activity among older adults with chronic conditions, especially cancer, we developed a mobile-enabled Web app called iCanFit [10] based on intensive formative research. We conducted in-depth interviews, surveys, and group discussions with older cancer survivors (60 years and older), care providers, and community leaders. They identified lack of motivation, lack of tracking, inadequate social support, and limited knowledge of appropriate exercise as the main barriers to regular exercise [11]. These formative data informed the design of the iCanFit Web application, which includes 4 key functions: Goals (physical activity goal setting and tracking), Community (an online network for users), Tips (regularly updated tips on healthy living), and Resources (active links to reliable health information) (see Figure 1 for screenshots of iCanFit). Note the Goals and Community functions in iCanFit are only available to registered users because the program is currently under efficacy trial. Of these functions, Goals is the most important tool (Figure 2) because it motivates participants to exercise regularly through goal setting, activity tracking, personalized feedback, and progress reviews. After a participant creates an account and logs onto the site the first time, s/he is invited to set a long-term goal; for example, “Over the next 6 months, I will go from walking 3 times a week to walking 5 times a week.” Participants are then asked to set a short-term goal, usually a weekly goal. They can use dropdown menus to select a type of activity (eg, walking, dancing), frequency per week, and duration of the activity. The system will automatically calculate total minutes for each activity and all activities (Figure 2). On an interactive calendar, participants can enter their activity and log the total number of minutes they exercised on a selected day (Figure 3). Their activity log will be compared to their goals and they will receive tailored messages based on this comparison; for example, “Congratulations, you’ve achieved your goal, keep up the good work!” or “Sorry you did not meet your goal. You may consider setting a more realistic goal. Keep moving!” (Figure 3). The tailored message is sent automatically from iCanFit using a predesigned database that contains more than 100 messages for different conditions of meeting goals. Finally, View Progress allows users to track their progress through various metrics, including total energy expenditure (metabolic equivalent of task, also referred to as MET), total minutes exercised, number of days exercised, and comparisons between actual activity and their preset goals (Figure 4). For MET and total minutes exercised, users have the option to view their progress as bars, lines, and/or a calendar. Under the Days Exercised view, their activities are marked against the goals they set (Figure 4).

During the iCanFit protocol development, we conducted an iterative heuristic evaluation with experts from behavioral sciences, computer science, human factors and ergonomics, exercise sciences, public health, and gerontology. The goal of the current study was to test usability and acceptability of iCanFit among older adults.

## Methods

### Study Design Overview

The study took place in a college town of central Texas from April to June 2014. Following the usability testing methods proposed by Kushniruk et al [12] and Schneiderman [13], we aimed to test usability and acceptability of iCanFit. Usability measures technical effectiveness and efficiency. Technical effectiveness is measured by recording whether the users can complete a given task or not and if they completed the task, whether they did so without error. Efficiency measures how much time it takes to complete each task [12,13]. Acceptability measures users’ overall experience with an application, including perceived ease of use, usefulness of the information, and satisfaction of the experience [14,15]. Our plan was to make changes to iCanFit based on data from usability testing; after the changes were completed, we proceeded with acceptability testing.

Considering significant variance in computer skills of older adults, testing in a laboratory may not reflect users’ experience in a real-life setting [16,17]. Thus, we planned to conduct the usability and acceptability testing in a community setting. Finally, based on theories regarding an appropriate sample size for usability testing and prior studies [18-21], the sample size for usability testing was set at approximately 10 and the sample for acceptability testing was set at approximately 20.

Based on these theories and rationale, our testing of iCanFit was designed to include 2 phases. The first phase was usability testing in a computer room at a senior community center. The participants were given the name of the website and asked to explore the site on their own. During this phase, a research assistant (RA) measured and observed the user’s navigation using usability testing metrics (see Multimedia Appendix 1). Based on data from usability testing, the Web application was improved by removing bugs and refining to be more senior-friendly. The second phase was acceptability testing. The participants were instructed to use the iCanFit on their own devices at home for 2-3 weeks and then give feedback through a user-experience survey (see Multimedia Appendix 2).

### Testing Tools Development

We developed a protocol of usability and acceptability testing that guided every step of the process. The protocol included recruitment scripts, a recruitment flyer, usability testing metrics, and a user-experience survey. We conducted intensive training with RAs to ensure the testing protocol was followed with fidelity.

Usability testing metrics (see Multimedia Appendix 1) were developed based on prior testing metrics employed in usability testing of online programs and mobile health technologies [12,14,22,23]. The metrics included 15 tasks to complete navigation of the site and detailed observation metrics to measure technical effectiveness and efficiency (eg, how much time it takes to complete a task and what errors occur during the navigation). It also included space for the RA to document users’ comments and behaviors during the testing.

The user-experience survey (see Multimedia Appendix 2) was developed from the IBM computer user satisfaction questionnaire [24], which has been widely used in similar acceptability testing studies [14,20]. It is a semi-structured interview with 57 questions on users’ experience with iCanFit. The survey included the following major components: (1) modes and frequency of accessing iCanFit and time spent on the site, (2) Likert scales on ease of use (1=very difficult, 4=very easy) and perceived usefulness (1=useless, 4=very useful), (3) open-ended questions on users’ satisfaction with each function and overall experience with the Web application, and (4) users’ comments on how to improve iCanFit and suggestions on how to promote physical activity among older adults.

### Participant Recruitment

We recruited our participants through active community outreach. Flyers were posted at senior community centers and public libraries and announcements were made during breaks of classes or programs for seniors. Participants who were aged 60 years or older and had used the Internet were invited to participate in our study. A total of 33 participants were recruited for the study; 10 conducted usability testing in a computer room in a senior center and the remaining 23 participants performed acceptability testing by using iCanFit for 2-3 weeks at home followed by a user-experience survey. The study protocol was approved by the Institutional Review Board at the Texas A&M University.

### Usability Testing Procedure

In the first phase of usability testing, the participants were invited to a computer room in a senior center. The RAs first introduced themselves and explained the purpose and procedure of the testing. Participants were assured of their privacy and verbal consent was obtained. An RA was paired with a participant. There was enough space between desktop computers to ensure proper testing and observation. A brief survey on participant demographics was administered before the testing. The participant was then given the website name and asked to explore the site on their own. The RA sat behind the participant and gave no instruction to the participant unless the participant could not proceed after repeated efforts. The participant was also encouraged to make comments during the navigation. The RA recorded the participant behavior and comments using observation metrics and took detailed notes. The metrics included 15 tasks to complete on the website, time needed on each task, and if a task was performed without error, with error, or needed assistance. Each participant received a US $20 gift card as compensation for their participation.

### Acceptability Testing Procedure

In the second phase of acceptability testing, another sample of 23 participants was recruited to test iCanFit on their own devices independently. When participants responded to our flyer by calling or speaking to our RAs at community outreach, they were instructed to visit the iCanFit site and use it for 2 to 3 weeks. An interview was then scheduled at the participant’s convenience to solicit their feedback on the website, including how difficult was it to navigate the site, the usefulness of each function and the site in general, and how satisfied they were with the website. Some interviews were conducted in-person at a community center and some were conducted over the phone. Verbal consent was obtained before the interview and each participant received a US $20 gift card as compensation for their participation.

### Data Analysis

All data were saved and analyzed in SPSS 16.0 (SPSS Inc, Chicago, IL, USA) and descriptive statistics were used to explore the mean usability and acceptability scores. All text data were extracted from SPSS and entered into ATLAS.ti (Berlin, Germany) for further analysis. We identified the most frequently used phrases or keywords, and delineated a range of responses for each task on iCanFit and their overall experience.

## Results

### Participant Characteristics

As shown in Table 1, 10 participants completed the usability testing in the computer room of a senior center. They were aged between 60 to 78 years with a mean of 68 (SD 6.3) years; 7 participants were female and 3 were male. Seven participants had some college education and the remaining 3 had high school education or less. All participants used the Internet and their computer experience varied from 3 to 30 years. The most typical mode of Internet access was desktop (60%, 6/10), followed by laptop (30%, 3/10), and tablet (10%, 1/10). All participants owned smartphones, but most used them primarily for making phone calls, whereas some used them for checking emails or using apps.

**Table 1:** Characteristics of participants in usability testing and user-experience survey of iCanFit.

Characteristics		Utility testing sample (n=10)	User-experience survey sample (n=23)
**Age (years)**			
	Mean (SD)	67.60 (6.3)	67.6 (6.5)
	Range	60-78	60-82
**Gender, n (%)**			
	Male	7 (70)	4 (17)
	Female	3 (30)	19 (83
**Education, n (%)**			
	≤High school	3 (30)	6 (26)
	>High school	7 (70)	17 (74)
**Internet use (years)**			
	Mean (SD)	20.30 (10.5)	20 (8.0)
	Range	3-38	6-30
**Common mode of Internet access, n (%)**			
	Desk top	6 (60)	11 (52)
	Laptop	3 (30)	8 (35)
	Tablet	1 (10)	2 (8)
	Smartphone	0	1 (4)

A total of 23 participants completed the user-experience survey; their ages were between 60 to 82 years (mean 68, SD 6.5). Of these 23, 19 (80%) were female and 17 (74%) had more than high school education. Approximately 52% (11/23) participants used a desktop as their primary mode of Internet access, followed by laptops (35%, 8/23), and tablets (8%, 8/23).

### Usability Testing: Effectiveness and Efficiency

Table 2 reports technical effectiveness (whether participants were able to perform tasks without errors) and relative user efficiency (how much time needed to complete each task) from usability testing. All participants were able to complete the 15 tasks in an average of 31 minutes (range 22-40 minutes). For each task, the completion time varied from 0.7 to 9.2 minutes.

The most difficult task appeared to be creating an account. It took participants an average of 9.2 minutes to create a user account. In all, 40% (4/10) could perform this task without error, 40% (9/23) completed the task with error, and 20% (2/10) needed assistance. Only 30% (3/10) watched the instructional video under the Help tab or logged out of the site after they completed all other tasks.

**Table 2:** Usability testing results of iCanFit (N=10).

Task	Time to complete (min)		Perform without error, n (%)	Perform with error, n (%)	Need assistance, n (%)
	Mean (SD)	Range			
Find the website	2.4 (2.3)	1-3	6 (60)	2 (20)	2 (20)
Create an account	9.2 (5.2)	1-17	4 (40)	4 (40)	2 (20)
Log into the account	2.7 (4.1)	0.5-11	8 (80)	1 (10)	1 (10)
Find Healthy Tips and read it	1.1 (1.6)	0-5	8 (80)	1 (10)	1 (10)
Find Resources and read it	3.4 (6.3)	0-20	9 (90)	0	1 (10)
Find the Facebook account through the link on the site	1.1 (1.3)	0-3	7 (70)	1 (10)	2 (20)
Find the Goal Home	0.7 (0.6)	0-2	7 (70)	1 (10)	2 (20)
Set a long-term goal	2.1 (2.3)	0-7	6 (60)	1 (10)	3 (30)
Set a short-term goal	1.4 (1.3)	0-4	6 (60)	1 (10)	3 (30)
Track the short-term goal by entering physical activity	3.4 (3.8)	0-8	5 (50)	2 (20)	3 (30)
Enter physical activity without tracking the short-term goal	2.1 (1.7)	0-5	9 (90)	1 (10)	0
View physical activity progress through View Progress	0.9 (1.1)	0-3	9 (90)	1 (10)	0
Switch view modes in View Progress	0		10 (100)	0	0
Find help	1 (1)	0-2	3 (30)	0	0
Log out			3 (30)	1 (10)	6 (60)
Time to complete the entire site	31.4 (6.9)	22-40			

### Improving iCanFit After Usability Testing

Through the observations and participants’ comments we obtained from the usability testing, we were able to identify and make the necessary changes to the iCanFit application to improve usability and senior friendliness. For example, comments from some of the participants indicated that some fonts and icons needed to be changed to be more visible for older adults. To address the problems participants had with goal setting, some words were changed to avoid confusion. For instance, after setting a short-term goal, the “Add Activity” button was changed to “Save.”

We also added some hot buttons for frequently used functions. For example, the “Exercised Today?” button was created allowing participants to enter activity before going to Goals and the “Log out” button was placed in a more visible location. During usability testing, we also learned that some pages of iCanFit did not display well on Internet Explorer 7.0 or lower, so we modified our site to make it compatible with more browsers.

Two major complaints from the participants were that they did not know what to do after they went onto the website and that they had trouble creating an account. Therefore, we changed our instructional video and made 3 separate videos, ranging from 30 seconds to 2 minutes in length, and explained (1) what iCanFit is and how to use it, (2) how to create an account, and (3) how to use Goals so that users can easily find the help they need.

### Acceptability Testing: User Experience and Satisfaction

Table 3 shows results from the user-experience survey. Most participants learned about iCanFit from a flyer at community centers. They typically accessed the website through a desktop (44%, 10/23) or a laptop (30%, 7/23), and the rest used a tablet (17%, 4/23) or a smartphone (9% 2/23). Most participants accessed the website approximately once a week or less and reported spending an average of 21.6 (SD 4.0) minutes on the site in the past week.

**Table 3:** Results of user-experience survey (N=23).

Variable		n (%)	Mean (SD)
**Sources of knowing iCanFit website**			
	Flyer at community center	15 (65)	
	Email listserve	2 (9)	
	Friend/relative	5 (22)	
	Other	1 (4)	
**Mode of accessing iCanFit**			
	Desktop	10 (44)	
	Laptop	7 (30)	
	Tablets	4 (17)	
	Smartphone	2 (9)	
**How often use iCanFit**			
	<Once/week	9 (39)	
	Approximately once/week	9 (39)	
	2-3 times/week	3 (13)	
	4-5 times/week	1 (4)	
	Every day	1 (4)	
Total time on iCanFit in past week (minutes; range 0-60), mean (SD)			21.6 (4.0)
**Difficulty (range 1-4)** ^a^			
	Creating account		3.2 (0.4)
	Long-term goal setting		3.7 (0.2)
	Short-term goal setting		3.8 (0.3)
	Short-term goal tracking		3.3 (0.3)
	View progress		3.7 (0.2)
	Overall difficulty		3.6 (0.3)
**Usefulness (range 1-4)** ^b^			
	Instructional video		3.7 (0.3)
	Healthy tips		3.5 (0.2)
	Resources		3.2 (0.2)
	Facebook page		3.1 (0.3)
	Overall usefulness		3.4 (0.3)
**Communication of iCanFit**			
	Ever talked to family/friends about iCanFit	13 (56.5)	
	Would recommend iCanFit to family/friends	13 (56.5)	

^a^ Difficulty score: 1=very difficult, 2=somehow difficult, 3=somehow easy, 4=very easy.

^b^ Usefulness score: 1=useless, 2=a little useless, 3=somehow useful, 4=very useful.

When asked to rate how difficult it was to use each function, most participants reported no difficulty or little difficulty in completing the major functions of the site with a score range of 3.2 to 3.8 (1=very difficult, 4=very easy). The overall difficulty for the major function of Goals was rated 3.6 (SD 0.3). When asked to rate usefulness of each function, most participants gave a rating of 3.1 to 3.7 (1=useless, 4=very useful). Participants gave the iCanFit website an average overall usefulness rating of 3.4 (SD 0.3). More than half (57%, 13/23) of the participants had talked to their family or friends about the iCanFit program and the same number of participants would recommend iCanFit to their family or friends.

Approximately 30% (7/23) of participants reported preferring to track physical activity in Goals through the “Exercised Today?” hot button and only 9% (2/23) liked to track activity through Track Goals; 22% (5/23) indicated having no preference because both were easy to use (data not shown).

Our qualitative data from the user-experience survey revealed that with different prior online experiences and varying statuses of current physical activity, participants had different experiences with iCanFit. For some participants who exercised regularly, they felt that the program added little to their current life: “I am doing exercise regularly, and I have a pedometer.” A couple of participants who did not use the computer often made remarks such as: “I prefer hardcopies of goals that can be stuck to the refrigerator so I can check it easily.” Most participants, however, were very positive about their experience with iCanFit, making comments such as: “Super great program, keep it up,” “It increased my activity because I was trying to get 100% of my goals,” and “It’s great to see how many times I have exercised; it gave me a kick to get up and accomplish something every day.”

Participants also offered suggestions on how to use mobile tools for older adults. For example, a 71-year-old male user commented, “It has to be something automatic or very easy to use. I like the dropdown menu when entering activities so I don’t need to type.” A 65-year-old female user added, “It would be nice if it has function to remind me to exercise, since we don’t remember things well at this age. And I like the graphs to see my progress.” They also shared thoughts on how to motivate older adults to exercise regularly. For instance (shared by a 68-year old female user), “If we can get people to start a program that combines a fitness class and how to use this site (iCanFit), you can motivate many sedentary people.” A 62-year-old male user suggested, “For those living alone, it is more about helping them find friends and getting them involved.”

## Discussion

Older adults from the community were recruited to test the usability and acceptability of a Web application designed to promote physical activities for older cancer survivors. Usability and acceptability was tested in settings familiar to the participants and on devices they often used to maximize their real-life experience. The relative user efficiency data, such as time to complete the tasks and errors made in first-time use, were within an acceptable range and reflected the anticipated usability gap between expert and novice users [22]. During the usability testing, the main challenge for users was account creation. Such a challenge might be because participants were new to the website and not aware that they needed to create an account before the major functions could be used. Some users did not know they would need to check their email to retrieve a password when setting up an account. These challenges and other errors identified through the usability testing were corrected and the site was further improved following users’ suggestions. The acceptability testing revealed a high level of ease of use and usefulness of iCanFit. Further, most participants reported they would continue using the program or recommend it to their families and friends.

As the use of mobile tools continues to increase, especially among older adults, mobile technology is being used increasingly as an efficient tool for health promotion [8]. Meanwhile, as the aging of the US population continues to accelerate, the need for cost-effective tools to address older adults’ health needs increases as well. We need more online or mobile programs designed for older adults, especially those with chronic conditions [8]. When developing and testing Web or mobile applications for older adults, the heterogeneity of this population should be considered because some seniors are savvy or expert users, whereas others are still new to computers or other mobile tools. Prior research indicates that many older adults are eager to obtain authoritative up-to-date health information and are willing to overcome barriers if appropriate assistance is offered [8,9,25]. This underscores the importance of designing age-appropriate programs for older adults.

In addition, when developing mobile programs for older adults, it is important to involve end users from early stage of design and conduct on-going usability testing [15,25]. From testing usability and acceptability of iCanFit among older adults, we learned that when conducting usability testing among older adults it is more efficient to start with some expert users. After critical errors are fixed, the application can be further tested with more typical users [15]. Finally, when testing acceptability and user experience, it is important to include clear instructions on what is being tested and how to use the application in the Web or mobile format, preferably by utilizing step-by-step instructions with pictures or video demonstrations.

Several limitations of the study should be noted. First, iCanFit was originally designed to promote physical activity among older cancer survivors [11] and usability and acceptability testing might be restricted to this population. However, the participants who completed the usability and acceptability testing in our study were older adults with a variety of chronic conditions, including cancer survivors. This expansion reflects the reality that the majority of older cancer survivors have existing comorbidities. Another reason for utilizing a less disease-specific user participant group was that we intend to expand the use of iCanFit to all older adults and our data have showed high levels of usability and acceptability of iCanFit among older adults. Second, we had a convenience sample recruited from a small city in central Texas, and most of our participants were white, female, had some college education, and were experienced with computers. The results may not be generalizable to older adults in other geographic locations or cultural settings. Third, because of the small sample size in the user-experience survey, we were not able to do comparisons between subgroups; for instance, differences in user satisfaction stratified by age, gender, computer skills, and chronic conditions. Future research should include a larger sample size and longer testing time to maximize end users’ inputs in site development. Finally, although we used mixed methods in the study, the qualitative questions were imbedded in a semi-structured interview and most users only provided short answers to those questions, thus giving us only limited qualitative data. Future research needs to include some in-depth interviews to explore the specific reasons users had for liking or disliking the application.

Despite these limitations, to the best of our knowledge, our study was one of the first to report testing of usability and acceptability of a Web app to promote physical activity among older adults. Our findings underscore the importance of using validated metrics and mixed methods to test multidimensional usability and acceptability of an application. An efficacy trial of the iCanFit Web application and development of iCanFit mobile app are both currently underway. After the trial and further refinement, it will be scaled up to assist a large population of older adults with chronic conditions. We are aware that there are many mobile and Web apps that serve similar purposes as iCanFit and users always have many options in terms of mHealth tools. iCanFit was not meant to replace existing physical activity applications; instead, we believe it is a beneficial supplement to the existing ones. Because few mobile or Web apps involved usability testing in older adults [15,25,26], our study represents an effort to voice older adults’ needs in the rapidly growing field of mobile health. We anticipate that in the near future, mHealth tools such as iCanFit will be more widely used by older adults to improve their healthy living.

## Appendix

Multimedia Appendix 1 iCanFit usability testing metrics.

Multimedia Appendix 2 iCanFit user-experience interview guide.

## Abbreviations

MET: Metabolic equivalent of task
RA: research assistant
