# Supplementary material for: Testing Usability and Acceptability of a Web Application to Promote Physical Activity (iCanFit) Among Older Adults
Source: JMIR Hum Factors. 2014 Oct 13;1(1):e2. doi: 10.2196/humanfactors.3787 (PMC4797672; doi:10.2196/humanfactors.3787)
Supplement: Multimedia Appendix 1 [file humanfactors_v1i1e2_app1.pdf]

ICANFIT usability testing metrics
www.icanfit.org

User demographics:

Age_______________ Gender________________
Education__________________ Years of using Internet______________
Typical mode of Internet access_____________________________

	Task	Perform without error	Perform with error	Need assistance	Other remarks
Account set up	Find the website				
	Create an account				
	Log into the account				
Healthy tips	Find Healthy Tips and read it				
Resources	Find Resources and read				
Community	Find the Facebook account through the link on the site				
Goal setting & tracking	Find the Goal Home				
	Set a long-term goal				
	Set a short-term goal				
	Track the short term goal by entering physical activity				
	Enter physical activity without tracking the short-term goal				
	View physical activity progress through View Progress				
	Switch view modes in View Progress				
Account set up	Log out				

Other observation items:
1. Watch instructional video? Yes______No______Remarks:____________________________
2. Time to navigate the website___________________
3. Other comments____

____
