# Supplementary material for: Testing Usability and Acceptability of a Web Application to Promote Physical Activity (iCanFit) Among Older Adults
Source: JMIR Hum Factors. 2014 Oct 13;1(1):e2. doi: 10.2196/humanfactors.3787 (PMC4797672; doi:10.2196/humanfactors.3787)
Supplement: Multimedia Appendix 2 [file humanfactors_v1i1e2_app2.pdf]

ICANFIT user experience survey

I. Use of ICANFIT

1. Where did you hear about ICANFIT website?
   a) Flyer at the community center
   b) Community newspaper
   c) Email listserve
   d) From a friend/relative
   e) Other_________________

2. How long have you used ICANFIT website? (check the account registration and compare)
   a) Less than 1 week
   b) 1 week-2 weeks
   c) 2-3 weeks
   d) More than 3 weeks

3. When you logged into the ICANFIT program, what device (computer, phone, tablet, etc.) did you use most often?
   a) Desktop computer
   b) Laptop
   c) Tablets (iPad, Galaxy, hp TouchPad, Dell tablet, etc.)
   d) Smart Phone or cell phone (iPhone, Samsung, Android phone, Nexus, etc).

4. How often did you log into ICANFIT?
   a) Less than once a week
   b) About once a week
   c) About 2-3 times a week
   d) About 4-5 time a week
   e) Every day

5. How much time did you spend on ICANFIT last week? ______minutes/week

6. Did you watch instructional video when you just entered the website?
   a) Yes
   b) No
   c) Don't remember

7. How many times did you watch it? _______________ Times

8. How useful was the instructional video?
   a) Useless
   b) A little useful
   c) Somehow useful
   d) Very useful
   e) I am not sure

9. What's your comments on the instructional video? (if did not watch, ask why)

____

____

10. When did you create a user's account on ICANFIT?
    a) The first time I went to the site
    b) The second time
    c) The third time
    d) After the 3rd time

11. How easy was it to create an account on ICANFIT?
    a) Very difficult
    b) Somehow difficult
    c) Somehow easy
    d) Very easy

12. What's your comments on creating an account? ________________________________

    ____

    ____

13. Have you used Goals in ICANFIT website?
    a) Yes
    b) No

14. How many times have you used the Goals function? __________________Times

15. Have you set a long-term goal in "Goal Setting)?
    a) Yes
    b) No

16. How easy was it to set a long-term goal?
    a) Very difficult
    b) Somehow difficult
    c) Somehow easy
    d) Very easy

17. Have you set a short-term goal in Goal Setting?
    a) Yes
    b) No

18. How many short-term goals have you set? ________________

19. How easy was it to set a short-term goal?
    e) Very difficult
    f) Somehow difficult
    g) Somehow easy
    h) Very easy

20. What's your comments on the Goal Setting function? (If didn't use it, ask why)___________

____

____

21. Have you entered physical activity through “Track Goals”?
    a) Yes
    b) No

22. Have you entered physical activity through “Exercise Today”?
    a) Yes
    b) No

23. How often did you enter physical activity?
    a) Almost every day
    b) Every 2-3 days
    c) Every 3-4 days
    d) Once a week
    e) Less than once a week

24. How easy was it to enter physical activity?
    i) Very difficult
    j) Somehow difficult
    k) Somehow easy
    l) Very easy

25. Which method do you prefer to enter physical activity?
    a) Through “Exercise Today”
    b) Through “Track Goals”
    c) Both are good
    d) Neither is good

26. What’s your comments on the Track Goals function? (If didn’t use it, ask why)___________

____

____

27. What’s your comments on the Exercise Today function? (if didn’t use it, ask why)______

____

____

28. Have you viewed your physical activity progress through “View Progress”?
    a) Yes
    b) No

29. Have you viewed your physical activity progress at the following modes in “View Progress” (Check all that apply)?
    a) METS
    b) Minutes Exercised

c) Days Exercised
d) None

30. (If ever viewed METS), which mode of view do your like most:
    a) Bars
    b) Lines
    c) Calendar
    d) None

31. (If ever viewed total minutes exercised), which mode of view do your like most:
    a) Bars
    b) Lines
    c) Calendar
    d) None

32. (If ever viewed Days Exercised), which mode of view do your like most:
    a) Boxes
    b) Bars
    c) Neither

33. How easy was it to use View Progress?
    a) Very difficult
    b) Somehow difficult
    c) Somehow easy
    d) Very easy

34. What's your comments on View Progress? (if didn't use it, ask why)___________________

____

____

35. How useful was the "Goals setting and tracking" for you?
    a) Useless
    b) Somehow useful
    c) Very useful
    d) I am not sure

36. What's your comments on Goals overall (if didn't use it, ask why)______________________

____

____

37. Have you ever visited Healthy Tips on the ICANFIT website?
    a) Yes
    b) No
    c) Don't remember

38. How many times have you checked Healthy Tips? ___________________Times

39. How useful was the Healthy Tips?
    a) Useless
    b) A little useful
    c) Somehow useful
    d) Very useful

40. What's your comments on the Healthy Tips? (if didn't use it, ask why)_____________

____

____

41. Have you ever visited Resources on the ICANFIT website?
    a) Yes
    b) No
    c) Don't remember

42. How many times have you checked Resources? __________________Times

43. How useful was the Resources?
    a) Useless
    b) A little useful
    c) Somehow useful
    d) Very useful

44. What's your comments on the Resources (if didn't use it, ask why)? __________________

____

____

45. Have you ever visited ICANFIT Facebook page? You can link it through "Community" in the tools?
    a) Yes
    b) No
    c) Don't remember

46. How many times have you visited the ICANFIT Facebook page? _________________Times

47. How useful was the Facebook page?
    a) Useless
    b) A little useful
    c) Somehow useful
    d) Very useful

48. What's your comments on the Facebook page? (if didn't use it, ask why)_______________

____

____

49. Overall, how useful was ICANFIT in promoting your physical activity?
    a) Useless
    b) A little useful
    c) Somehow useful
    d) Very useful

50. Can you talk more on usefulness of ICANFIT? (did it increase your physical activity, how much? Did it improve your quality of life? Etc)
    ____

    ____

51. Have you ever talked to your family or friends about ICANFIT?
    a) Yes
    b) No

52. Would you recommend ICANFIT to your friends and family?
    a) Yes
    b) No
    c) I Don't know

53. When you talked to your family and friends about ICANFIT, How did you talk to them and what were their reactions? ____

    ____

    ____

54. Do you have suggestions on how to motivate older adults to exercise more?_______

    ____

    ____

55. Do you have other questions or comments for us? _______________________________

____

____

56. Would you like to see a summary of the results of our ICANFIT project?
    a) Yes
    b) No

57. If yes, how do you like to receive the summary report?
    a) Email
    b) Mail
